# An observational study of the impact of service use on suicidality among adults with mental disorders

**DOI:** 10.1186/s40621-014-0029-9

**Published:** 2014-11-20

**Authors:** Guilherme Borges, Ricardo Orozco, Joshua Breslau, Matthew Miller

**Affiliations:** 1 National Institute of Psychiatry, Calzada México Xochimilco 101, Mexico City, Mexico; 2 RAND Corporation, Pittsburgh, PA USA; 3 Director, Undergraduate Program in Health Sciences, Northeastern University, Bouvé College of Health Sciences, Department of Health Sciences, Room 316 Robinson Hall, 360 Huntington Avenue, Boston, MA 02115-5000 USA; 4 Director, Harvard Injury Control Research Center, Adjunct Professor of Epidemiology, Harvard School of Public Health, Boston, MA USA

**Keywords:** Suicidality, Suicidal ideation, Suicidal attempts, Mental health service, Mental disorders, Treatment

## Abstract

**Background:**

It is unclear whether treatment of mental disorders reduces the probability that a) people without suicidal ideation will begin to contemplate suicide, or b) people who have thought about killing themselves (but have not attempted suicide) will go on to make an attempt.

**Methods:**

Mental disorders, service use for emotional or substance use problems, and suicidality were assessed using the World Mental Health version of the Composite International Diagnostic Interview. Discrete-time survival analysis was used to establish the temporal priority of mental health service use and suicide-related outcomes among the 5,862 participants in the Collaborative Psychiatric Epidemiological Surveys who reported a mental disorder.

**Results:**

Use of specialty mental health services, but not other types of services for emotional or substance use problems, was associated with an increased risk of future suicide ideation (OR = 1.27, CI = 1.01–1.60). However, respondents with a history of suicidal ideation were less likely to report a subsequent suicide attempt if they had received any type of service for emotional or substance use problems (OR = 0.62, CI = 0.46–0.83), regardless of the type of service received (i.e., it did not matter whether the service received was mental health care, general medical care, or non-health care related).

**Conclusions:**

Among persons with frank DSM disorders and suicidal ideation, the receipt of treatment is associated with a lower rate of subsequent suicide attempts, compared with those who never received treatment, regardless of treatment provider type. Follow-up studies are a logical next step to our observational investigation.

## Background

It has been reported that two-thirds of patients who attempt suicide have seen a general practitioner within the month prior to the attempt (Houston et al. [2003]), that repetition of attempts among patients treated in clinical settings is common (Yip et al. [2011]), and that death by suicide after an attempt varies from as low as 1.7% among respondents in community samples (Kuo and Gallo [2005]) to a high 13% among patients treated in hospitals who were followed for 37 years (Gibb et al. [2005]; Haukka et al. [2008]; Suominen et al. [2004]). The high rate of treatment among persons who died by suicide and among patients with repeated attempts does not speak one way or another to the benefits, with respect to suicide prevention, of treatment for mental disorders since these observations alone do not assess what the rate of suicide or repetition of self-harm might have been in the absence of treatment (i.e., no comparison is made to the frequency of these behaviors among comparable untreated persons). In fact, no prospective population based longitudinal study, and little empirical evidence more broadly, addresses whether traditional or non-traditional treatment for mental health disorders appreciably affects the risk of subsequent suicide or suicidal behavior for most at risk patients.

Cross sectional population surveys in the US (Kessler et al. [2005a]) and around the globe (Bruffaerts et al. [2011]) have documented suggestive but inconclusive observations about persons with lifetime suicide attempts and/or thoughts having commonly received some form of self-reported mental health treatment during their lifetime. Here too, however, it is not clear what impact such treatment may have had, nor, critically, whether suicidality preceded or followed treatment for mental disorders. Indeed, because few studies have established the temporal sequence of first treatment and the onset, alleviation, or worsening of suicidality, it remains unclear whether treatment affects the likelihood of transitioning from ideation to attempts —and if so, in what direction and to what extent. With few exceptions (Hegerl et al. [2006]), the role of treatment in the initiation, persistence, recurrence and extinction of suicidality among those with a mental disorder is largely unknown (Borges et al. [2008]; Kessler et al. [2005b]; Kuo et al. [2001]).

The current study addresses this gap in the literature by focusing on whether self-reported receipt of treatment for “problems with emotions, nerves, mental health, or use of alcohol or drugs” among those with a mental disorder is associated with transitioning from never having suicidal ideation to having ideation, and among those with ideation prior to treatment, from not having plans or making attempts, to having plans and making attempts. We also examine whether the strength of observed associations between treatment and suicide related outcomes depends on a) the provider sector, and b) the underlying broad category of mental disorder. We pursue these aims in a large general population sample of the US using data from a multi-ethnic national sample.

## Method

Data come from the National Institute of Mental Health (NIMH) funded Collaborative Psychiatric Epidemiological Surveys (CPES), which includes the National Comorbidity Survey Replication (NCSR), the National Latino and Asian-American Survey (NLAAS) and the National Study of American Life (NSAL) (Heeringa et al. [2004]). The CPES surveys were conducted during the same time period, using the same diagnostic instrument, and by the same field staff certified by the Institute for Social Research at the University of Michigan (Alegría et al. [2007]; Kessler and Merikangas [2004]; Takeuchi et al. [2007]; Williams et al. [2007]).

The instrument used in these surveys, the World Mental Health version of the Composite International Diagnostic Interview (WMH-CIDI) was developed with NIMH support to assess DSM-IV mood, anxiety and substance use disorders, as well as suicidality and a broad range of suspected risk factors. The CIDI is a fully structured computer assisted diagnostic interview. After complete description of the study to the subjects, informed consent was obtained. CPES data from 5862 respondents with a history of mental disorders were used and categorized for this study into four suicidality groups. IRB from the National Institute of Psychiatry in Mexico City approved this analyses.

### Measures of suicide-related outcomes

The WMH-CIDI contains a module that assesses history of suicide ideation (“Have you ever seriously thought about committing suicide?”), suicide plan (“Have you ever made a plan for committing suicide”) and suicide attempts (“Have you ever attempted suicide?”). These questions were printed in a booklet and referred to in the interview by letter given that evidence suggests participants’ reports of such potentially sensitive behaviors are higher in self-administered than in interviewer-administered surveys (Turner et al. [1998]). Respondents with a history of any suicide ideation, plan or attempt were asked the age at which they first experienced that outcome. Timing of prior suicide ideation, plan and attempt was obtained using a question sequence shown experimentally to improve recall accuracy (Knäuper et al. [1999]). We considered four main groups: 1) those without any ideation or suicidal behavior; 2) those who reported suicide ideation only, 3) those who reported ideation and a plan only, 4) those who reported ideation and a suicide attempt with or without a plan.

### Psychiatric disorders

Psychiatric disorders were diagnosed according to Diagnostic and Statistical Manual of Mental Disorders, Fourth Edition (DSM-IV) criteria. The common diagnostic assessment in all surveys included mood disorders (major depressive disorder and dysthymia), anxiety disorders (panic disorder, agoraphobia without panic disorder, social phobia, generalized anxiety disorder and posttraumatic stress disorder) and substance use disorders (alcohol abuse, drug abuse, alcohol dependence with abuse, and drug dependence with abuse). All respondents were asked the age at which they had first experienced any of those disorders.

### Demographics

Multivariate analyses also used sociodemographic information including gender, age, educational attainment, age at first marriage and divorce (if any), nativity (US born Vs foreign born) and race/ethnicity. Race/ethnicity was coded in broader ethnic groups: Asian, Black, Hispanic, White and Other (Collaborative Psychiatric Epidemiology Surveys [2008]).

### Service use

Respondents were asked about lifetime receipt of services for emotional, alcohol, or drug problems and the type of provider from whom services were received. Using methods described elsewhere (Bruffaerts et al. [2011]; Kessler et al. [2005a]; Wang et al. [2007]) mental health care service providers were divided into the following five types: 1) psychiatrists; 2) other mental health specialists, consisting of psychologists, counselors, psychotherapists, mental health nurses, and social workers in a mental health specialty setting; and 3) general medical practitioners, consisting of family physicians, general practitioners, and other medical doctors, such as cardiologists, or gynecologists (for women) and urologists (for men), nurses, occupational therapists, or other health care professionals; 4) human services, including outpatient treatment with a religious or spiritual advisor or a social worker or counselor in any setting other than a specialty mental health setting, or a religious or spiritual advisor, such as a minister, priest, or rabbi; 5) complementary-alternative medicine including Internet use, self-help groups, any other healer, such as an herbalist, a chiropractor, or a spiritualist, and other alternative therapies. For this study, provider types were collapsed by service sector into mental healthcare (types 1 and 2), general medical (type 3) and the non-health care sector (types 4 and 5).

### Analysis

After estimating the lifetime prevalence of suicidal behavior and service use by suicidal ideation, plan and attempts in the CPES, discrete-time survival analysis with time-varying covariates (Efron [1988]; Willett and Singer [1993]) was used to examine the associations between service use and further risk of suicidality, adjusting for sociodemographic variables. Discrete-time survival analysis was used instead of common logistic regression to model the first occurrence of suicide-related outcomes as this allows for incorporation of the temporal ordering of onset of service use and suicidality, time-varying sociodemographics (e.g., marital status, educational attainment), and mental disorders. We used weights developed by CPES biostatisticians for the sample to be representative of the general US population (National Institute of Mental Health [2010]). In these models, the independent variable is the age of onset of service use (any, and by type of provider) and the outcome variables are a) the age of first onset of suicide ideation (in the total population) and b) among those with a suicide ideation the age of first onset of a suicide plan and a suicide attempt.

Survival coefficients in this discrete-time survival analyses model were converted to odds ratios (ORs) for ease of interpretation; significance tests of sets of coefficients in the logistic regression models were made using Wald *X*^2^ tests based on design-corrected coefficient variance-covariance matrices. These ORs are interpreted as the increase in the risk of suicidality (ideation in the total sample or plan and attempt among those with an ideation) after the use of services for the treatment of mental disorders and substance use problems. We estimated standard errors by the Taylor series method with SUDAAN version 10.0.1 (National Institute of Mental Health [2010]; Research Triangle Institute [2009]) to adjust for the weighting and clustering of the data. We also report 95% confidence intervals (CIs) adjusted for design effects, stratification, and clustering and for unequal weighting of the observations.

## Results

Among the 5,862 respondents with a history of mental disorders, most were females (54.3%), 34.8% were 18–35 years old, 77.3% whites, 9.8% Blacks, 10.6% Hispanics, and 2.3% of Asian origin. Most respondents, 69.8% of the sample, were free of any lifetime suicidality; lifetime prevalence of suicide ideation only was 14.4%, lifetime plan only was 5%, and 10.8% reported an attempt (4% without a plan, 6.8% with a plan). These prevalences tended to be modestly larger among females.

### Service use and suicidality total and by provider

Table 1 presents the prevalence of service use for the treatment of mental disorders across categories of suicidality among those with mental disorders. Lifetime use of services among those with suicidality was common, and for every subgroup, suicidality increased the probability that a subject would report having used health and non-health care services. The lifetime prevalence of any service use was 63.5% in the absence of a suicide ideation, with service use increasing to 78.9% in the presence of suicide ideation. The presence of a plan further increased the use of services to 84.5% and among those with an attempt, with and without a plan, 88.4% reported using services. Specialty mental health care was always the most commonly used type of service.

**Table 1:** **Lifetime service use among categories of suicidal behaviors, by type of provider, in a Collaborative Psychiatric Epidemiology Surveys, among respondents with a mental disorder**

		Any service		Any mental healthcare		General medical		Non-healthcare	
Lifetime	Frequency	n	% (SE)	n	% (SE)	n	% (SE)	n	% (SE)
Non suicidal	4138	2526	63.52 (1.44)	1639	40.50 (1.16)	1514	36.28 (1.14)	1078	25.36 (1.12)
Ideation only	794	615	78.90 (1.63)	468	58.53 (2.07)	365	46.69 (2.83)	290	34.62 (1.94)
Ideation with plan only	255	206	84.53 (3.05)	164	69.09 (4.38)	125	51.20 (3.10)	116	43.30 (3.70)
Ideation with attempt	675	596	88.37 (1.51)	527	77.23 (2.10)	321	45.43 (1.75)	313	45.69 (2.76)
***X***^2^ (p-value)*	-	216.2 (<0.001)		245.2 (<0.001)		59.27 (<0.001)		84.58 (<0.001)	

*Chi-square test on main categories (three degrees of freedom).

Frequencies are unweighted; percentages are weighted.

### The impact of service use in the progression of suicidality

Among those with lifetime suicidality and service use, service use was more commonly initiated after the onset of suicidal ideation (39%) than before the onset of ideation (31%) or in the same year as the onset of ideation (30%). However, service use was more common before a plan or an attempt (about 39%), than in the same year (31%) or after (30%).

Table 2 shows multivariate models for the first occurrence of ideation in the sample of persons with DSM disorders and the first occurrence of a plan or an attempt in the subsample of ideators. The use of any services was associated with a non-significant increase of subsequent suicide ideation. Those that used services from “any mental healthcare” provider showed an increased risk of ideation (OR = 1.27, 95% Confidence Interval 1.01–1.60), particularly among those with an anxiety or substance use disorder.

**Table 2:** **Risk of suicidal behaviors after treatment for mental disorder or substance use disorder, by type of provider, in the Collaborative Psychiatric Epidemiology Surveys, overall and by group of disorders**

		Any service		Any mental healthcare		General medical		Non-healthcare	
	n	OR	95% CI	OR	95% CI	OR	95% CI	OR	95% CI
Model 1. Ideation in the total population	5245	1.18	(0.94–1.46)	1.27	(1.01–1.60)	1.05	(0.76–1.45)	0.95	(0.62–1.47)
i. Among people with mood disorder	2219	0.98	(0.73–1.33)	1.02	(0.76–1.36)	0.85	(0.57–1.28)	0.81	(0.50–1.32)
ii. Among people with anxiety disorder	3256	1.27	(0.98–1.65)	1.39	(1.06–1.82)	1.01	(0.69–1.49)	0.93	(0.57–1.52)
iii. Among people with substance use disorder	1570	1.37	(1.00–1.88)	1.48	(1.00–2.21)	1.38	(0.85–2.24)	1.10	(0.67–1.81)
Model 2. Plan with ideation and no lifetime attempt	954	0.89	(0.58–1.36)	0.92	(0.52–1.64)	1.20	(0.70–2.05)	0.67	(0.26–1.75)
i. Among people with mood disorder	533	0.93	(0.49–1.78)	0.96	(0.49–1.85)	1.01	(0.44–2.36)	0.68	(0.18–2.60)
ii. Among people with anxiety disorder	633	0.86	(0.51–1.45)	0.76	(0.47–1.22)	1.22	(0.64–2.35)	0.87	(0.36–2.09)
iii. Among people with substance use disorder	319	0.94	(0.56–1.59)	1.33	(0.49–3.59)	1.48	(0.61–3.57)	0.32	(0.10–1.01)
Model 3. Attempt with ideation	1513	0.62	(0.46–0.83)	0.67	(0.49–0.92)	0.68	(0.46–1.00)	0.56	(0.35–0.90)
i. Among people with mood disorder	811	0.91	(0.58–1.44)	0.91	(0.59–1.42)	0.92	(0.52–1.64)	1.07	(0.52–2.17)
ii. Among people with anxiety disorder	1043	0.64	(0.44–0.93)	0.73	(0.49–1.09)	0.54	(0.32–0.91)	0.65	(0.36–1.18)
iii. Among people with substance use disorder	494	0.80	(0.43–1.48)	0.85	(0.44–1.64)	1.19	(0.55–2.57)	0.74	(0.39–1.43)

Each row and column is one model with suicidal behavior as dependent variable and type of provider as independent variable, adjusted by sex; age four categories: 18–25, 26–35, 36–45, 46–89; education four categories: 0–11, 12, 13–15, 16+ years of education; marital status three categories: married/cohabiting, divorced/separated/widowed, never married; race/ethnicity four categories: Asian, Hispanic, Black, White and other; US born (yes/no). Model 3 is also adjusted by presence of a plan and age of onset of a plan. Person-time is accounted for in all models.

Among those with suicide ideation prior to first service use, however, the use of services (any provider, any mental health provider, and any non-healthcare provider) was associated with a non-significant lower likelihood of developing a suicide plan (ORs ranging from 0.67 to 0.92) (Model 2, Table 2). Moreover, there was a significantly lower likelihood of reporting a subsequent suicide attempt (OR = 0.62, CI = 0.46–0.83), regardless of provider group (ORs ranging from 0.56 to 0.68) (Model 3, Table 2). Additional analyses by type of mental disorders showed few instances of interaction, the only significant finding being that those with anxiety disorders showed a reduced risk of attempts if they used any service or a general medical provider (OR = 0.64 and OR = 0.54, respectively).

Sensitivity analyses produced findings in line with primary analyses. The number of service providers and the type of provider had little impact on the association between service use and suicide related outcomes. For example, those subjects reporting that they had seen one provider only were less likely to report subsequent suicide attempts than those seeing no service provider, OR =0.60 (95% CI = 0.38–0.95), as were, to a similar extent, those reporting having seen exactly 2 providers, OR =0.50 (0.29–0.86), and those reporting having seen up to 5 providers, OR = 0.38 (0.054–2.61). In addition, our findings did not materially change when we accounted for the number of disorders as an indirect measure of the severity of mental health disorders.

## Discussion

Self-reported receipt of treatment for “problems with emotions, nerves, mental health, or use of alcohol or drugs” was significantly associated with increased suicide ideation only if respondents used a mental health provider. By contrast, among persons with suicidal ideation, subsequent suicidal behavior (plan and attempt) was more likely among those who did not receive services for emotional, alcohol or drug problems, compared with those who received services. Interestingly, the type of service use (mental health care vs. general medical care vs. non-health care) and the type of mental disorder had little impact.

Associations between service use and the lack of recovery from suicide ideation has been reported before (Gunnell et al. [2004]), as has an increased risk of suicide re-attempt among attempters who used mental health services (Murphy et al. [2012]). To our knowledge, however, no study prior to the current one has examined whether service use is associated with the *new* onset of suicidality among people with a psychiatric disorder. It is possible that our finding of higher rates of new onset suicide ideation among persons who self-reported receipt of treatment with a mental health care provider is due, in part or in whole, to confounding by indication, with those who have more severe disorders being both more likely to develop suicide ideation (Gunnell et al. [2004]) and more likely to turn to specialized services. It is not clear why we found higher risk of new onset suicidal ideation among those with anxiety and substance use disorders, but not mood disorders.

Our main finding that treatment for emotional, alcohol or drug problems was associated with less frequent development of subsequent suicide attempts is qualified in that it is unclear why the three types of service use (mental health care vs. general medical care vs. non-health care) appear to provide similar advantages. Indeed, we did not find that the medical sector had a higher impact on suicidality than the non-healthcare sector, or that the use of a mental health specialist had a larger impact than the use of the general medical sector. With respect to this finding, again it is possible that we are facing an example of confounding by indication (Moscicki et al. [1988]; Murphy et al. [2012]; Psaty et al. [1999]): patients with more severe disorders may be more likely to consult with a psychiatrist, thereby lowering the apparent impact of treatment when compared with less seriously afflicted persons consulting with a general medical practitioner or with non-health practitioners. Unfortunately, we do not have measures of disorder severity to help sort out this possibility. Sensitivity analyses of our results using the number of disorders (comorbidities) as proxy for severity did not, however, substantially change our findings.

Our main results are consistent with prior research that shows treatment for mental disorders may lower the prevalence of suicide plans and attempts (Hegerl et al. [2006]), but less so for persons who only screen positive for these disorders (Patel et al. [2011]) or who have less severe depression (Alexopoulos et al. [2009]). Lowering suicidality among persons treated for substance use disorders has been reported as well (Britton and Conner [2010]; Ilgen et al. [2007]), but defining the best providers (Simon and Savarino [2007]) and the best pharmacological or psychological treatments to prevent repetitions of suicide attempts (Daigle et al. [2011]) has been elusive. Overall, our results point to an area in which further studies are needed, such as those that follow patients prospectively, with better information on the level of suicidal intent, severity of psychiatric disorders, and with specific details about the treatment delivered and how well patients adhere to treatment plans.

Our findings should be viewed with other limitations in mind as well, chief among which is that our analyses are based on retrospective self-reports, which may be affected by recall bias if some groups are systematically more likely to accurately recall and report past life events or treatment episodes than others. Recall bias is a potential problem especially among the elder population of our survey. The data on service use, for example, are based solely on self-report. In the absence of confirmatory information on treatments, we cannot assess the validity of these data, or the possibility that mental disorders produce differential recall of service use. Second, the single CIDI questions used to elicit suicide ideation, plan and attempt are clearly limited and did not include any measure of chronicity or severity of suicide ideation or attempt. Furthermore, no reliability or validity data were obtained for measures of ideation, plan or attempt, restricting inferences that can be drawn from this study. Clinical studies of suicidal patients usually include a much more in-depth evaluation of the patient’s state of mind and collect more granular information about suicide risk factors and treatments prescribed (Mann et al. [1999]; Oquendo et al. [2005]). Such follow-up studies are a logical next step to our observational investigation. Our CIDI questionnaire, while comprehensive for most common mental disorders, did not include psychotic and other infrequent mental disorders in all CPES surveys (e.g., bipolar disorder). It would be unusual for these disorders to be present in our study subjects in the absence of the other common comorbid disorders we account for explicitly. Third, we had to limit the number of mental disorders included in our estimations, since not all the surveys endorsed the same DSM-IV diagnoses and we included only those common to all survey instruments used, thus underestimating the prevalence of disorders reported here. Fourth, we have no information about what kinds of treatment were received in the past within each service use category and therefore cannot address, for example, whether some types of mental health treatments (talk therapy or medication) are more helpful (or more harmful) than others.

## Conclusions

Despite these limitations, this large epidemiological study of 5,862 respondents with a history of mental disorders suggests that treatment for DSM disorders was significantly associated with increased suicide ideation only if respondents used a mental health provider, and among persons with suicidal ideation treatment is associated with a lower rate of subsequent suicide attempts, regardless of treatment provider type. It is not clear why provider type –mental health, general medical, or non-health care related –all appear to provide similar benefit.

## Abbreviations

NIMH: National Institute of Mental Health
CPES: Collaborative Psychiatric Epidemiological Surveys
NCSR: National Comorbidity Survey Replication
NLAAS: National Latino and Asian-American Survey
NSAL: National Study of American Life
WMH-CIDI: World Mental Health version of the Composite International Diagnostic Interview
DSM-IV: Diagnostic and Statistical Manual of Mental Disorders, Fourth Edition
